# GRP78 expression in peripheral blood mononuclear cells is a new predictive marker for the benefit of taxanes in breast cancer neoadjuvant treatment

**DOI:** 10.1186/s12885-020-06835-z

**Published:** 2020-04-19

**Authors:** Annat Raiter, Julia Lipovetzki, Ido Lubin, Rinat Yerushalmi

**Affiliations:** 1 Felsenstein Medical Research Center, Sackler School of Medicine, Tel Aviv University, Rabin Medical Center, Beilinson Campus, 49100 Petach Tikva, Israel; 2 Davidoff Cancer Center, Rabin Medical Center, Beilinson Campus, 49100 Petach Tikva, Israel

**Keywords:** GRP78 expression, Breast cancer, Neoadjuvant chemotherapy, Peripheral blood mononuclear cells, Interferon gamma

## Abstract

**Background:**

Breast cancer treatment is tailored to the specific cancer subtype. Often, systemic treatment is given prior to surgery. Chemotherapy induces significant endoplasmic reticulum (ER) stress-mediated cell death and upregulation of 78-kDa glucose-regulated protein (GRP78). We hypothesized that chemotherapy induces ER stress not only in the tumor tissue but also in immune cells, which may affect the response to anti-cancer treatment.

**Methods:**

We determined the surface expression of GRP78 on 15 different peripheral blood mononuclear cell (PBMC) subpopulations in 20 breast cancer patients at three time points of the neoadjuvant treatment, i.e., at baseline, after anthracycline treatment, and after taxanes treatment. For this purpose, we performed flow cytometric analyses and analyzed the data using ANOVA and the Tukey test. Serum cytokine levels were also evaluated, and their levels were correlated with response to treatment using the *t*-test after log transformation and Mann-Whitney U Wilcoxon W test.

**Results:**

A significant increase in GRP78 expression in PBMCs was documented during the taxane phase, only in patients who achieved pathological complete response (pCR). GRP78-positive clones correlated with increased serum levels of interferon gamma (IFNγ).

**Conclusions:**

The presence of GRP78-positive clones in certain PBMC subpopulations in pCR patients suggests a dynamic interaction between ER stress and immune responsiveness. The correlation of GRP78-positive clones with increased levels of IFNγ supports the idea that GRP78 expression in PBMCs might serve as a new predictive marker to identify the possible benefits of taxanes in the neoadjuvant setting.

## Background

Breast cancer is the most common cancer in women worldwide and the second leading cause of cancer death in women. In 2018, over two million new cases were reported by the World Health Organization [1]. There are various subtypes of breast cancer, such as luminal A, luminal B, (human epidermal growth factor receptor) 2) Her2^+^, and triple-negative being Estrogen receptor (ESR), Progesterone receptor (PR) and Her2 negative [1, 2]. Because breast cancer is an extremely heterogeneous disease, medical oncologists try to tailor the treatment for each patient to the specific subtype. One of the current strategies is to use a neoadjuvant regimen, a systemic treatment that is provided prior to surgery. Such a strategy enables actual validation of the potential benefit from the treatment in real time [3]. Therefore, the neoadjuvant setting represents an appealing platform to study biomarkers that may predict benefit from a specific agent [4].

Nowadays, an increasing number of patients are subjected to systemic treatment prior to surgery as part of the standard of care or a clinical trial [5]. This is considered a window of opportunity for treatment optimization [6]. Most of the patients who need chemotherapy in this setting will be offered a regimen that contains anthracyclines and taxanes [7, 8]. Although these two agents are considered fundamental in breast cancer treatment, they are beneficial only in some patients and are associated with high toxicity [9, 10]. The magnitude of response to neoadjuvant treatment strongly correlates with patient prognosis after surgery; an optimal result is achieved when no residual invasive disease is detected and is determined as pathological complete response (pCR) [11, 12].

It has been well established that chemotherapy acts, at least in part, by triggering endoplasmic reticulum (ER) stress [13]. Many chemotherapeutic drugs, such as doxorubicin [14] and paclitaxel [15], used in the neoadjuvant setting in breast cancer, and cisplatin [16], oxaliplatin [14], and 5-fluorouracil [17], induce massive ER stress-mediated cell death. Paclitaxel, in particular, induces other ER stress-related pathways, including the upregulation of 78-kDa glucose-regulated protein (GRP78) [18]. When ER stress occurs, the unfolded protein response (UPR) is activated to minimize ER stress-associated injuries [19]. Under stress conditions, GRP78, a key molecule in the UPR, dissociates from the UPR sensors protein kinase RNA-like endoplasmic reticulum kinase (PERK), inositol-requiring enzyme 1 (IRE1), and activating transcription factor 6 (ATF6), and becomes activated and overexpressed [20]. It has been shown that elevated expression of GRP78 in tumors leads to its translocation to the cancer cell surface [21].

In the last decade, the search for new cancer biomarkers has focused on tumor protein and gene expression, serum soluble proteins, circulating tumor cells, and circulating free DNA [22]. Despite all efforts, most patients receive similar chemotherapy regimens, mainly because biomarker profiling has not led to a panel that effectively enables protocol tailoring [23, 24].

We recognized that the tumor microenvironment is an important participant in immune activation and response to treatment. The UPR modulates cytokine production through inflammatory signaling pathways and the regulation of cytokine transcription factors [13, 25]. Based on those and previous findings in our laboratory [26, 27], we hypothesized that chemotherapy may induce ER stress not only in the tumor tissue, but also in immune cells, which affect the response to the treatment. Therefore, in this study, we focused on the ER stress response in leucocyte subpopulations exposed to neoadjuvant breast cancer treatment. We evaluated cell surface expression of GRP78 in different peripheral blood mononuclear cell (PBMC) subpopulations of patients with breast cancer before, halfway, and at the end of the neoadjuvant treatment. In addition, we measured inflammatory and immune-suppressor factors in the serum. We found GRP78-positive clones in certain PBMC subpopulations in patients who achieved pCR. Additionally, we observed a correlation between the GRP78-positive clones and increased levels of IFNγ in serum of pCR patients, suggesting a dynamic interaction between ER stress and immune responsiveness.

## Methods

### Patients

The protocol study was approved by the Institutional Review Board at Rabin Medical Center, Israel (0667–14-RMC). Informed written consent was obtained from each patient who participated in this research. The cohort investigated included patients who were diagnosed with breast cancer at the Rabin Medical Center and were treated with neoadjuvant systemic chemotherapy. The patients were referred to surgery according to the standard of care. Healthy women (*n* = 10) were recruited randomly as a control group.

The neoadjuvant regimen consisted on anthracyclines and taxanes. Four cycles of intravenous (i.v.) doxorubicin and cyclophosphamide (AC) every 2–3 weeks, followed by 12 weeks of i.v. paclitaxel were administered prior to surgery. In patients with triple negative breast cancer, carboplatin was added to the taxanes phase. In case of Her2^+^ disease, trastuzumab and pertuzumab (monoclonal antibodies targeting Her2) were added to the taxanes phase.

Blood and serum were collected from each patient at three time points: Just before the first AC administration, before any treatment (baseline; P1), 2) after four cycles of AC and just before commencing the paclitaxel phase (P2) 3) Just before the last paclitaxel administration (end of treatment; P3). Serum was stored at − 70 °C until use. Blood was immediately processed for fluorescence-activated cell sorting (FACS) analysis.

To investigate the correlation between chemotherapy-induced stress on the immune system and treatment outcome, data from the final pathological reports in the breast and lymph nodes were retrieved. Patients were categorized into two groups according to the report: those with pCR and those with residual disease (non-pCR). pCR was defined as disappearance of any invasive disease. Patients that did not achieved pCR (non pCR) were those with residual component of any invasive disease.

### FACS analysis of cell surface GRP78 expression in PBMC subpopulations

Blood (200 μl/tube) was transferred to FACS tubes. The cells were incubated with fluorochrome-conjugated antibodies (Beckman Coulter, Brea, CA, USA) that identify the different PBMC subpopulations and the anti-GRP78-Bi DyLight 488 mouse monoclonal antibody (Abcam, Cambridge, UK) [28], as summarized in Table 1. To ensure specificity, a Fluorescence Minus One **(**FMO) control was used to identify and gate cells in the context of data spread due to the presence of multiple fluorochromes in any given panel. In brief, for each sample, two tubes were used: one tube containing cells stained with the antibodies identifying the different PBMC subpopulations and an isotype control for the anti-GRP78 antibody (IgG2a-DyLight 488, Abcam), and one tube containing cells stained with the antibodies identifying the different PBMC subpopulations and with the anti-GRP78 antibody. The percentage of cells expressing GRP78 on the cell surface in each subpopulation was calculated after subtracting the fluorescence obtained for the control tube. After staining, the erythrocytes were lysed in 1 mL of BD FACS lysing solution (BD Biosciences, San Jose, CA, USA) at 20–25 °C for 15 min. The cells were washed with phosphate-buffered saline (PBS) and suspended in 0.5 mL PBS for FACS (Coulter Navios flow cytometer, Indianapolis, IN, USA). Data were analyzed with the Kaluza software (Beckman Coulter, IN, USA).

**Table 1:** Schematic representation of the antibodies added to the different FACS tubes for the determination of GRP78+ clones

Tube 1a control	Tube 1b	Tube 2a control	Tube 2b
Anti-CD3-PC5.5	Anti-CD3-PC5.5	Anti-CD3-PC7	Anti-CD3-PC7
Anti-CD4-PC7	Anti-CD4-PC7	Anti-CD14-KrO	Anti-CD14-KrO
Anti-CD8-KrO	Anti-CD8-KrO	Anti-CD62L-PB	Anti-CD62L-PB
Anti-CD56-PE	Anti-CD56-PE	Anti-CCR7-PE	Anti-CCR7-PE
Anti-CD16-AF 750	Anti-CD16-AF 750	Anti-CD45RA-APC-AF 750	Anti-CD45RA-APC-AF 750
Anti-NKG2D-APC	Anti-NKG2D-APC	Anti-CD45RO-PC5.5	Anti-CD45RO-PC5.5
IgG2a-DyLight 488	Anti-GRP78-DyLight 488	IgG2a-DyLight 488	Anti-GRP78-DyLight 488

### Determination of inflammatory and immune-suppressor cytokines in the serum

Cytokines concentration in patient serum was evaluated with the MILLIPLEX MAP Human High Sensitivity T Cell Panel - Immunology Multiplex Assay (Merck KGaA, Darmstadt, Germany), which quantifies interleukin (IL)-2, IL-4, IL-6, IL-10 IL-23, tumor necrosis factor alpha (TNFα), and interferon gamma (IFNγ), following the manufacturer’s instructions.

### Statistical analysis

Results are presented as the mean ± standard error (sem). Mean cell surface expression of GRP78 was compared among the different PBMC subpopulations using one-way ANOVA and the Tukey test. The results obtained in patients with pCR were compared with those from patients with non-pCR using the *t*-test after log transformation and the Mann-Whitney U Wilcoxon W test. The Wilcoxon W test is a nonparametric test used to determine whether two dependent samples were selected from populations having the same distribution.

Cytokine secretion data in the pCR and non-pCR groups were compared using ANOVA with repeated measures and time as the within-patient factor. Serum cytokine levels were correlated with response to treatment using the *t*-test after log transformation and Mann-Whitney U Wilcoxon W test.

All statistical tests were two-sided, and *P* < 0.05 was considered significant.

## Results

### Clinicopathological characteristics of the patients

Thirty women (twenty breast cancer patients and ten healthy women) were recruited in this study. Of the twenty patients, three were classified as ESR^+^/Her2^−^, eleven as Her2^+^, and six as having triple-negative tumors. Patient characteristics are presented in Table 2.

**Table 2:** Patient characteristics

Pathological response	All patients (pCR + Non-pCR)	pCR	Non-pCR
**Age (years)**			
Median (range)	61.7 (33–69)	65.3 (38–69)	58.1 (34–68)
**Subtype**			
TN	6	3	3
Her2^+^	11	9	2
ESR^+^/Her2^-^	3	2	1
**Tumor size stage**			
T0	1	1	0
T1	13	10	3
T2	1	0	1
T3	5	3	2
**Nodal stage**			
N0	5	3	2
N1	10	9	1
N2	2	2	0
N3	3	2	1
**Total**	20	14	6

*ESR* Estrogen Receptor, *pCR* pathological Complete Response, *TN* triple negative

### Cell surface expression of GRP78 in PBMCs

We first observed that the percentage of T, NK and monocytes sub-populations vary between PBMCs of breast cancer patients before and after chemotherapy. However, the variations between the different individuals were not statistically significant (Table S1).

**Fig. 1:**
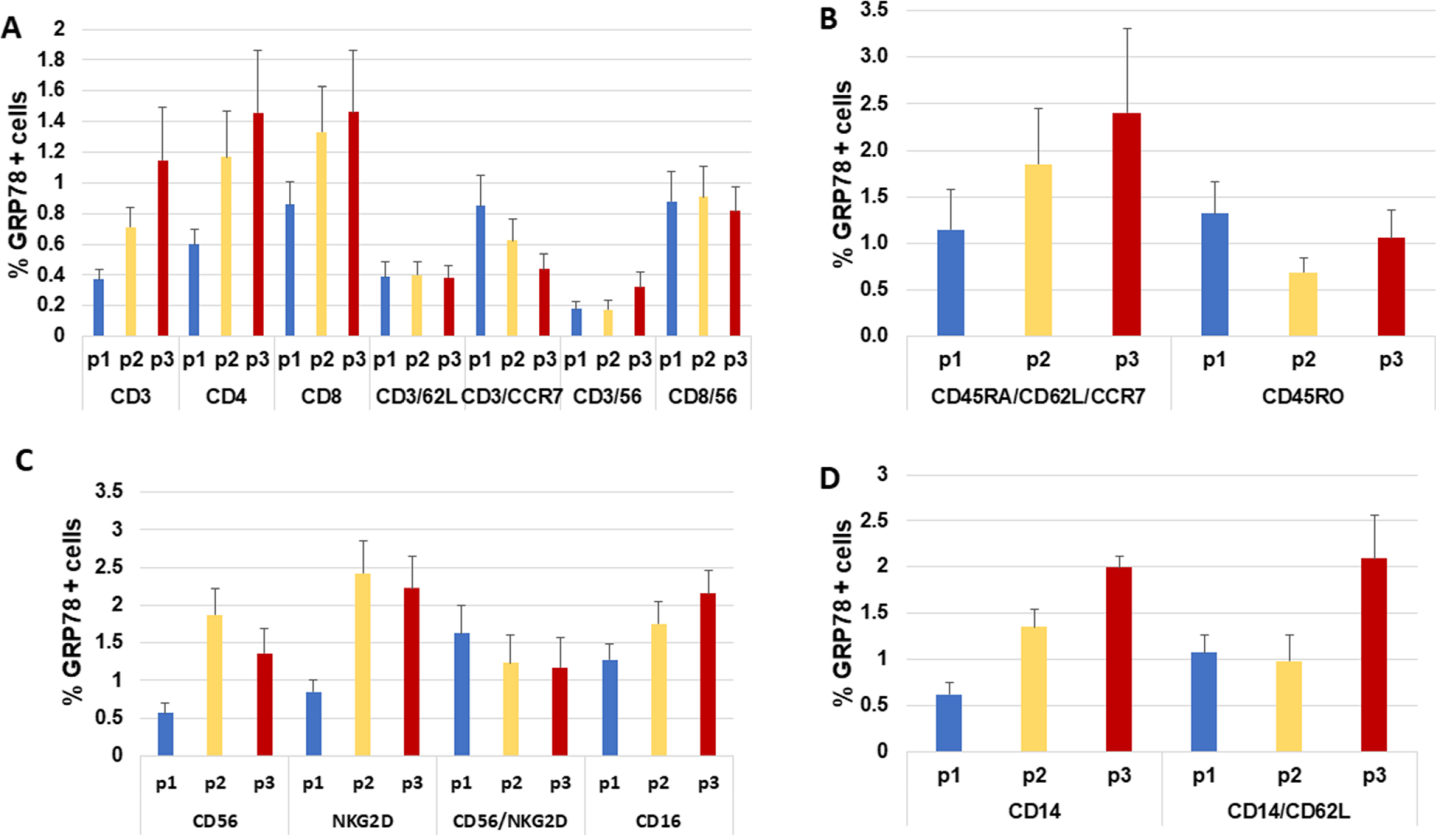
Surface GRP78 expression in PBMC subpopulations. GRP78 expression was determined by FACS in 15 different PBMC subpopulations at three time points in the neoadjuvant setting: P1 (prior to any treatment), P2 (after the AC phase), and P3 (after taxane phase). Mean cell surface expression of GRP78 was compared among different PBMC sub-populations using one-way ANOVA and Tukey tests. **a** T cell subpopulations; **b** T memory cells; **c** natural killer cells; **d** monocytes

We then, determined the baseline (P1) GRP78 expression in 15 different PBMC subpopulations derived from patients with breast cancer prior to any treatment and in healthy women. The percentage of the GRP78 positive sub-populations is a fraction, where the denominator is the whole population. Among the different PBMC subpopulations from the patients, we identified specific clones that expressed GRP78. Cell surface GRP78 expression varied from 0.19% ± 0.14% CD3^+^/CD56^+^ cells to 1.58% ± 0.38% in CD56^+^/NKG2D^+^ cells (Fig. 1a and c). The following PBMC subpopulations presented > 1% GRP78 expression: CD56^+^/NKG2D^+^, CD16^+^ (1.32% ± 0.2%), CD45RA^+^/CD62L^+^/CCR7^+^ (1.1% ± 0.43%), and CD45RO^+^ (1.21% ± 0.49%, Fig. 1b). In contrast, cell surface GRP78 expression was absent in the different PBMC subpopulations isolated from healthy women.

### Effect of treatment on GRP78 surface expression

The effect of cancer neoadjuvant therapy on ER stress was evaluated by determining the expression of GRP78 on PBMC subpopulations at P2 (AC phase) and P3 (paclitaxel phase) as indicated in Fig. 1. The AC phase (P2) induced surface GRP78 expression in some PBMC subpopulations. GRP78 expression in CD4^+^ T cells increased from 0.58% ± 0.1% (at P1) to 1.17% ± 0.3% (at P2; Fig. 1a). A non-significant increase (from 1.1% ± 0.4 to 1.9% ± 0.6%) in GRP78 expression was observed in CD45RA^+^ T cells (which were also positive for CD62L and CCR7) (Fig. 1b). In the natural killer (NK) subpopulation (CD56^+^), GRP78 expression increased from 0.55% ± 0.3 to 1.83% ± 0.34% and in NKG2D^+^ cells, it increased from 0.84% ± 0.16 to 2.3% ± 0.44% (Fig. 1c). Chemotherapy also affected GRP78 expression in CD14^+^ cells, where it increased from 0.6% ± 0.1% to 1.35 ± 0.2% (Fig. 1d).

The paclitaxel phase (P3) induced a significant increase in cell surface GRP78 in the CD3^+^ subpopulation. GRP78 expression in CD3^+^ cells increased from 0.37% ± 0.07% (at P1) to 1.15% ± 0.38%, *P* < 0.02 (Fig. 1a). The impact of paclitaxel was observed also in naïve memory cells (CD45RA^+^/CCR7^+^/CD62L^+^, Fig. 1b), in which GRP78 expression increased from 1.1% ± 0.43 to 2.4% ± 0.9% and in CD14^+^/CD62L^+^ cells, in which it increased from 1.02% ± 0.19 to 2.1% ± 0.47% (Fig. 1c and d); however, these effects were not significant.

### GRP78 expression in PBMCs from patients of the pCR and not-pCR groups

**Fig. 2:**
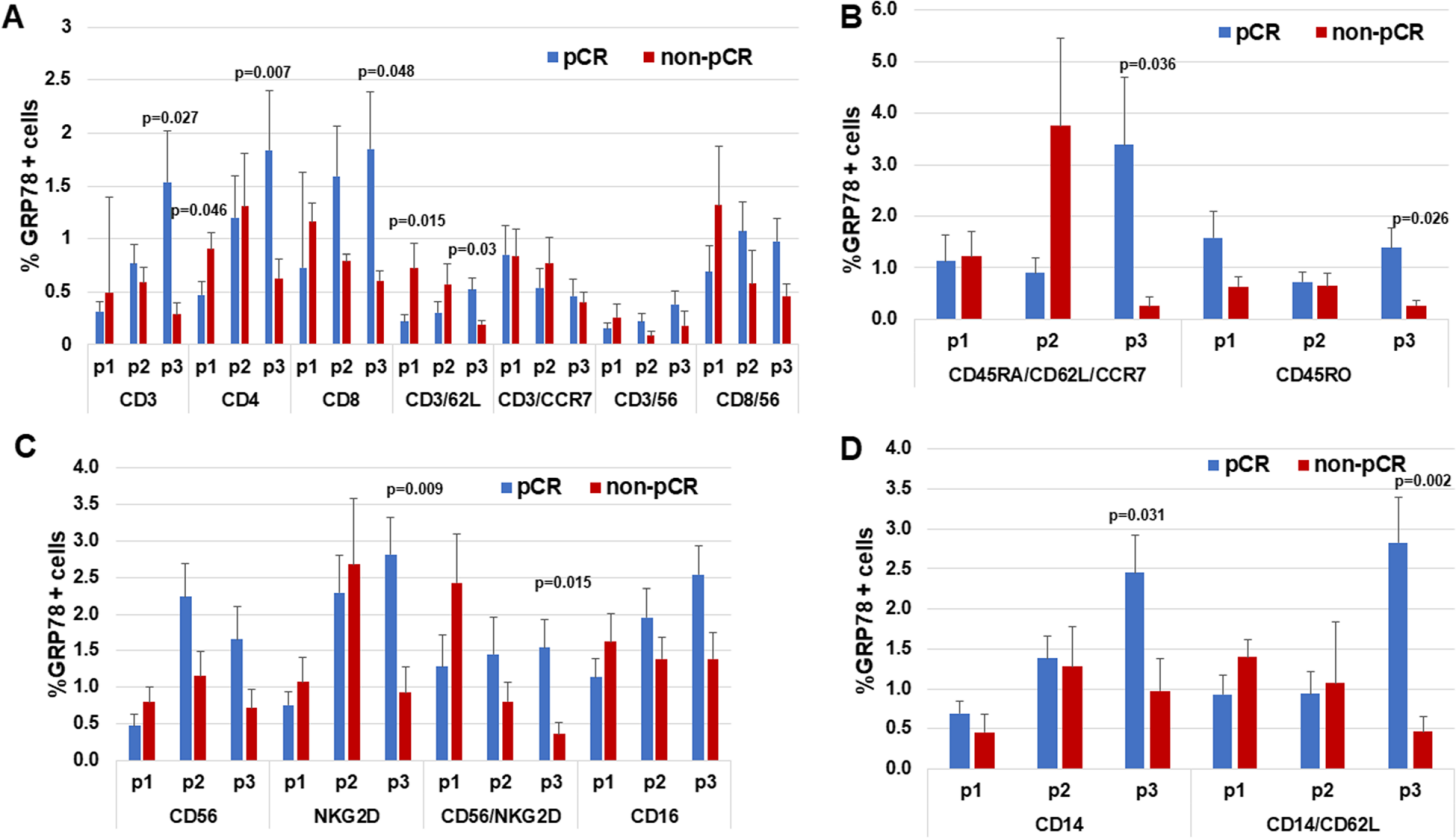
Comparison of GRP78 expression in PBMC subpopulations from patients of the pCR and non-pCR group. Data for pCR and non-pCR patients were compared with the *t*-test after log transformation and Mann-Whitney U Wilcoxon W tests. GRP78 expression at baseline (P1), after AC treatment (P2) and after taxanes treatment (P3) was evaluated by FACS on different PBMCs patients’ subpopulations. **a** T cells, **b** T memory cells, **c** NK cells and **d** monocytes

Forteen patients achieved pCR (disappearance of any invasive disease) and six patients had residual disease (non-pCR). Baseline GRP78 expression was similar in the two groups, with the exception of two subpopulations: GRP78 expression in CD4^+^ cells was significantly higher in the non-pCR group than in the pCR group (0.91% ± 0.15 and 0.45% ± 0.12% respectively, *P* = 0.046, Fig. 2a). CD3^+^/CD62L^+^ cells also demonstrated higher cell surface GRP78 expression in the non-pCR group (0.72% ± 0.2%) than in the pCR group (0.22% ± 0.06%, *P* = 0.015, Fig. 2a).

AC induced GRP78 expression in some PBMC subpopulations; however, the increase was not statistically significant. AC had no impact on GRP78 expression in CD8^+^, CD3^+^/CCR7^+^, and T memory cells (Fig. 2b).

The paclitaxel phase induced GRP78 expression in most PBMC subpopulations. Significant increases were observed in the following PBMC subpopulations of patients who achieved pCR compared to those who did not: CD3^+^ (*P* = 0.027), CD3^+^/CD62L^+^ (*P* = 0.032), CD4^+^ (*P* = 0.007), and CD8^+^ (*P* = 0.048) T cells (Fig. 2a); CD45RO^+^ effector T memory cells (*P* = 0.026) (Fig. 2b); CD56^+^ (Fig. 2c): NKG2D^+^ (*P* = 0.009) and CD56^+^/NKG2D^+^ (*P* = 0.015) NK cells (Fig. 2c); and CD14^+^ (*P* = 0.031) and CD14^+^/CD62L^+^ (*P* = 0.002) monocytes (Fig. 2d). Additionally, the percentage of cell surface GRP78 in naïve CD45RA^+^/CD62L^+^/CCR7^+^ cells increased significantly compared to P1 (*P* = 0.036).

**Fig. 3:**
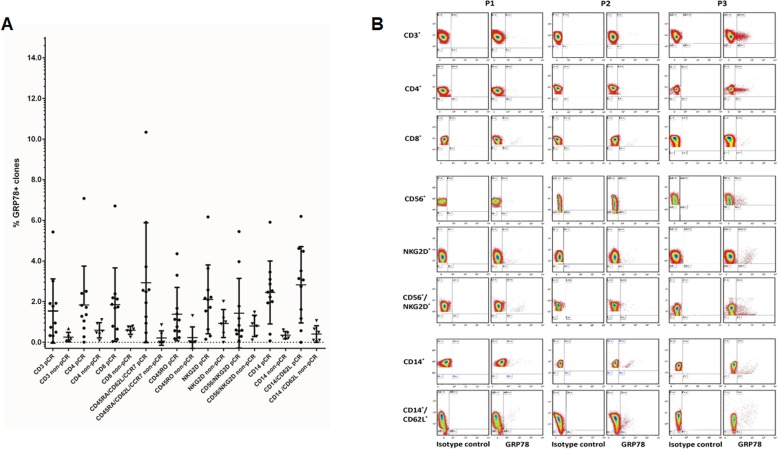
Comparison of individual pCR and non-pCR patients. **a** Cell surface GRP78 expression in PBMC subpopulations in each patient after the taxane phase (P3) comparing patients who achieved pCR with non-pCR patients. **b** Representative FACS data dot blot of one pCR patient showing the FMO control for each PBMC subpopulation at the baseline (P1), after AC treatment (P2), and after taxanes treatment (P3)

The percentage of PBMCs expressing GRP78 upon paclitaxel treatment for each individual patient is presented in Fig. 3a. A representative FACS data dot blot of a patient who achieved pCR is presented in Fig. 3b.

### Cytokine secretion in the serum of patients of the pCR and non-pCR groups

**Fig. 4:**
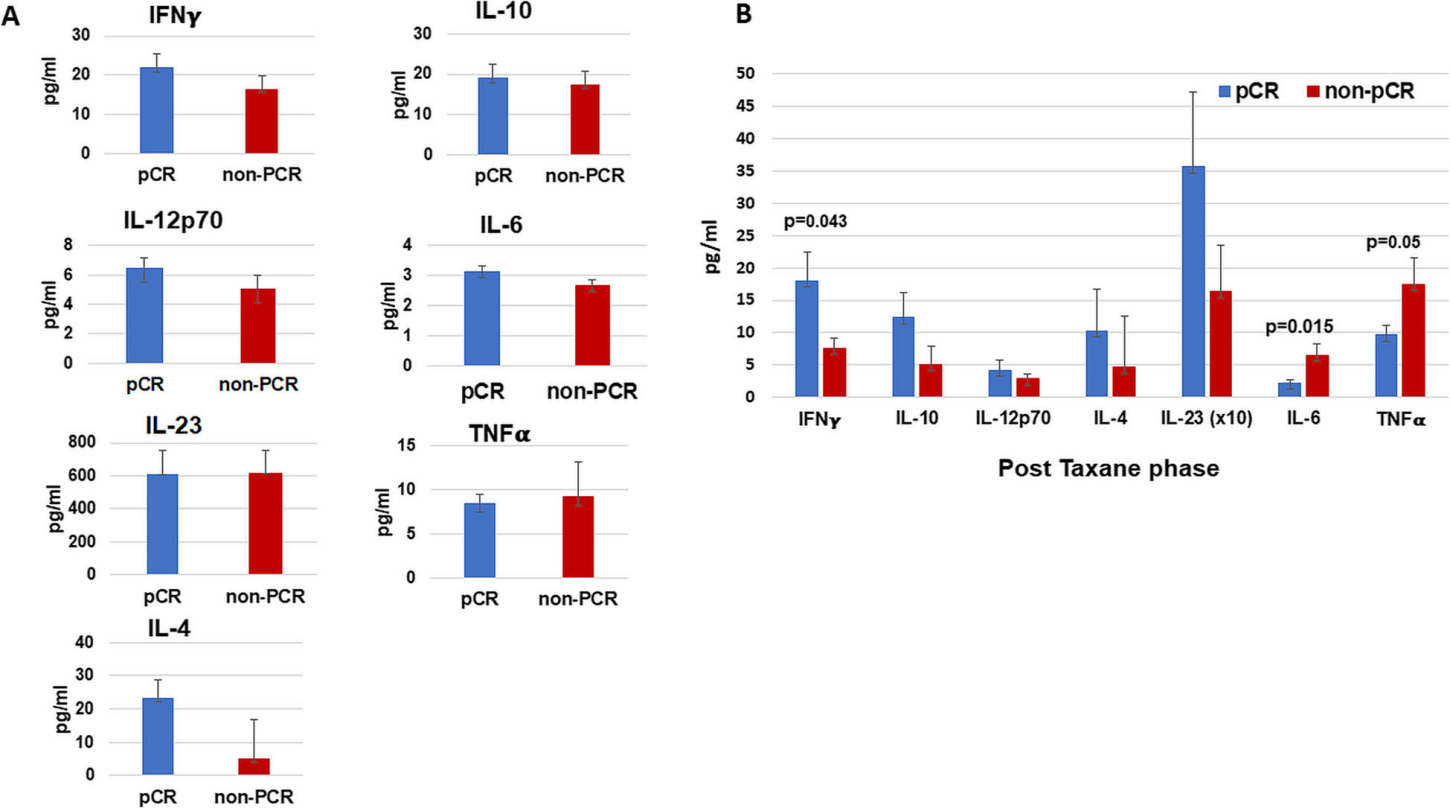
Serum cytokine secretion in pCR and non-pCR-patients. Cytokine secretion levels were compared in pCR and non-pCR patients using ANOVA. **a** Baseline (P1), **b** After the taxane phase (P3)

Serum levels of IFNγ, IL-10, IL-12p70, IL-4, IL-23, IL-6, and TNFα were evaluated in the patients at different time points (P1 and P3; Fig. 4). The baseline levels of all cytokines were similar in patients who achieved pCR and those who did not (Fig. 4a). After the taxane phase, IFNγ secretion was significantly higher in the pCR group than in the non-pCR (*P* = 0.043; Fig. 4b). No significant differences were found in IL-4 and IL-10, IL-12p70, and IL-23 levels after taxanes treatment, although the levels tended to be higher in patients of the pCR group. In contrast, IL-6 and TNFα levels after the taxane phase were significantly higher in non-pCR patients (*P* = 0.015 and *P* = 0.05 respectively).

## Discussion

The current study describes, for the first time, the effect of cancer therapy on the ER stress in immune cells subpopulations isolated from the immune system of patients with breast cancer. Specifically, we detail the changes in the cell surface expression of GRP78 in PBMC subpopulations isolated from patients undergoing neoadjuvant chemotherapy.

First, we observed, no statistical differences between the percentage of T, NK cells and monocytes sub-populations among the breast cancer patients before and after chemotherapy. We concluded, after extensive analysis, that the percentage of the subpopulations, have no impact on the cell surface GRP78 positive subpopulations.

We observed the presence of GRP78-positive clones in specific PBMC subpopulations of patients with breast cancer prior to any treatment. On the other hand, in healthy women, no surface GRP78 expression was observed in any of the PBMC subpopulations. Cell surface expression of GRP78 in PBMCs, such as mononuclear cells, has been reported in patients with rheumatoid arthritis [29]. In addition, GRP78 surface expression has been detected in CD4^+^ retrovirus-transduced murine cells, in which it forms a complex with latency-associated peptide and transforming growth factor beta (LAP/TGFβ) [30]. In contrast, other studies have rebutted the concept of cell surface GRP78 expression on lymphocytes [31, 32]. It is well known that breast cancer is a systemic disease, even in the early stages and it is reasonable to expect continuous interactions between immune cells and circulating tumor cells. We believe that the interaction between circulating tumor cells and immune cells leads to ER stress activation that consequently induces the surface expression of GRP78 in different PBMC subpopulations prior to any treatment [33].

Interestingly, GRP78 expression in the various PBMC subpopulations was different between the pCR and the non-pCR patients prior to any treatment. Specifically, two subpopulations, CD4^+^ and CD3^+^/CD62L^+^ cells, more strongly expressed GRP78 at the baseline in patients of the non-pCR group. This may suggest that different tumor and/or microenvironment characteristics and different interactions between immune cells such as CD4^+^ and CD3^+^/CD62L^+^ cells and tumor cells already exist at the beginning of the disease [34]. Notably, in the non-pCR group, the GRP78^+^ clones comprised less than 1 % of the entire immune cell population at the baseline and thus may not have a substantial effect.

In previous studies, including ours, it has been shown that the hostile tumor microenvironment causes chronic ER stress, which eventually leads to the elevation of GRP78 expression on the surface of tumor cells. Under these conditions, tumor cells activate the pro-survival component of the ER stress response system, suppressing apoptosis [18, 26]. Paclitaxel induces severe ER stress [15, 18] that overrides the protective ER stress response efforts and elicits its pro-apoptotic effect [35]. In this study, we demonstrated that, in addition to what is known about the effect of treatment-induced stress on the tumor, chemotherapy also affects the immune system as well.

After completion of the neoadjuvant regimen, we observed an increase in GRP78 expression in CD3^+^, CD4^+^, T memory cells (CD45RA^+^), NK cells (CD56^+^), activated NK cells (NKG2D^+^), and CD14^+^/CD62L^+^ in all patients, regardless of their response to treatment. A striking finding was the significant increase of GRP78 expression in specific PBMC subpopulations in patients who achieved pCR at the end of the taxane phase. Importantly, this increase was not observed at the end of the AC phase nor in the non-pCR group. This suggests that GRP78 might serve as a new predictive biomarker to predict the benefits from taxanes treatment in the neoadjuvant setting. The positive impact of leukocyte GRP78 expression in the pCR patients after taxanes treatment can be explained by the fact that the severe stress had activated CD4^+^ T cells, which led to augmented antitumor responses [36].

Different PBMC subpopulations of the pCR patients demonstrated significant increases in GRP78 expression. The subpopulations with less than 1 % of GRP78+ clones were T cells (CD3^+^/CD62L^+^), T naïve memory cells (CD45RA^+^/CD62L^+^/CCR7^+^), and T effector memory cells (CD45RO^+^). It should be mentioned that GRP78 expression in T naïve memory cells increased after the AC phase, whereas GRP78 expression on effector T memory CD45RO^+^ cells initially decreased after AC and significantly increased after taxane treatment. This was observed only in the pCR patients. In addition, NKG2D^+^ cells and CD14^+^/CD62L^+^ monocytes showed a significant increase in GRP78 expression in the same patient group. It has been well established that T cells and monocytes migrate to the lymph nodes via CD62L and chemokine receptor CCR7 [37, 38]; upon their differentiation into effectors, CD62L and CCR7 are downregulated in T memory cells, which then increasingly express CD45RO. Monocytes expressing CD62L preferentially migrate to the lymph nodes to induce adaptive immune responses against cancer cells [39]. The GRP78-positive clones in the effector T cells (CD45RO), activated NK (NKG2D^+^) cells, and monocytes migrating to the lymph nodes in the pCR patients after the taxane phase may be correlated with the immune response that eliminated residual cancer cells [40]. Altogether, these results suggest that GRP78 expression has a beneficial role in the neoadjuvant treatment.

To gain insight in the microenvironmental changes during the treatment, we evaluated changes in the secretion of specific stress-related cytokines. We found significantly higher concentrations of the inflammatory cytokine IFNγ in the sera of patients who achieved pCR only at the end of the taxane phase: IFNγ levels were correlated with the presence of GRP78-positive clones in PBMC subpopulations in these patients. Previous studies described anti-tumor CD8(+) T cell responses with increase in IFNγ levels in pCR Her2-positive breast cancer patients undergoing neoadjuvant chemotherapy [41]. Accumulating data show that UPR activation plays a role in a number of physiological events associated with immune cells and with modulation of inflammatory signaling pathways, resulting in cytokine production [42, 43]. The interaction between the UPR and factors secreted by the inflammatory process is a two-way pathway. Chemotherapy activates UPR, increasing cell-surface GRP78 in leukocytes which eventually leads to the secretion of inflammatory cytokines such as IFN-γ. IFN-γ has been described as a central orchestrator of antitumor immune responses [44]. The secreted inflammatory cytokines can induce ER stress, creating a positive feedback loop [45, 46]. We suggest that the modulation of cytokine production by endoplasmic reticulum stress during chemotherapy may be involved in the anti-tumoral immune response. This hypothesis is based on our detection of cell-surface GRP78 expression only in the PBMCs from patients who achieved pCR and the elevation in IFNγ in the serum from these patients’ leucocytes.

IL-6 and TNFα levels were significantly elevated in sera of non-pCR patients. Our results confirm those of a previous report demonstrating elevated levels of TNF-α and IL-6 in sera of patients diagnosed with advanced breast cancer and high load of metastases [47]. In a previous study using multivariate analysis of clinical prognostic parameters and cytokines, serum IL-6 was also identified as an independent adverse prognostic variable for overall survival [48]. In our study, two out of the six patients who did not achieve pCR and lacked GRP78-positive clones developed metastases shortly after completing the neoadjuvant treatment.

Unexpectedly, IL-23, IL-10, IL-12, and IL-4 concentrations were comparable in both the pCR and non-pCR groups. These cytokines are reportedly associated with ER stress and cancer progression [48]. However, in this study, no correlation between chemotherapy-induced ER stress or response to therapy and the above cytokines was found.

This study had some strengths as well as weaknesses. First, this is a prospective study in which all blood samples were drawn at exactly the same time points, as per the predesigned protocol.

Each patient served as her own control, making it possible to obtain statistically and clinically significant results despite the relatively small cohort. In addition, no patients were lost to follow-up. The results were obtained by FACS analysis using an in-house protocol that allows for concomitant analysis of GRP78 expression in 15 different PBMC subpopulations, including an internal control for each result.

The study also has some weaknesses. The main weakness of this study is the relatively small cohort analyzed and short follow-up. A longer follow-up would have made it possible to correlate the findings with patient overall survival and disease-free survival.

Conclusion: this study has revealed for the first time a correlation between the presence of GRP78-positive clones among effector immune cells of patients with pCR at the end of the neoadjuvant treatment, suggesting a dynamic interaction between ER stress and immune responsiveness. The increase in GRP78-positive clones at the end of a taxane phase in patients who achieved pCR suggests that surface GRP78 expression in PBMCs might serve as a new predictive marker of the benefit of taxanes. Larger studies are required to elucidate whether GRP78 expression in specific PBMC subpopulations can serve as a predictor marker for the benefit of different chemotherapies. Given the significant results, we believe that these findings may lead to the development of novel therapies to treat breast cancer based on the interaction between the immune system and ER stress.

## Supplementary information

**Additional file 1: Table S1.** Percentage T, NK and monocytes sub-populations before and after chemotherapy.

## Data Availability

The datasets generated for this study are available from the corresponding author on reasonable request.

## Abbreviations

ER: endoplasmic reticulum
GRP78: 78-kDa glucose-regulated protein
PBMC: peripheral blood mononuclear cell
pCR: pathological complete response
IFNγ: Interferon gamma
Her2^+^: human epidermal growth factor receptor
ESR: Estrogen receptor
PR: Progesterone receptor
PERK: RNA-like endoplasmic reticulum kinase
IRE1: inositol-requiring enzyme 1
ATF6: Activating transcription factor 6
AC: Adriamycin and cyclophosphamide
i.v.: intravenous
P1: baseline
P2: At the end of AC phase
P3: paclitaxel phase
FACS: fluorescence-activated cell sorting
FMO: Fluorescence Minus One
IL: interleukin
NK: Natural Killer
CD3: T cells
TNFα: tumor necrosis factor alpha
PBS: phosphate-buffered saline
sem: standard error
NKG2D: Activated NK
CD45RA^+^/CD62L^+^/CCR7^+^: Naïve T memory
CD45RO^+^: Effector T memory
CD4^+^: T helper
CD8^+^: T cytotoxic
CD14: Monocytes
CD62L: L-selectin
CCR7: C-C chemokine receptor type 7
UPR: unfolding protein response

## References

[1] Bellanger M, Zeinomar N, Tehranifar P, Terry MB (2018). Are global breast cancer incidence and mortality patterns related to country-specific economic development and prevention strategies?. J Glob Oncol.

[2] Ozturk K, Dow M, Carlin DE, Bejar R, Carter H (2018). The emerging potential for network analysis to inform precision cancer medicine. J Mol Biol.

[3] Rubovszky G, Horváth Z (2017). Recent advances in the neoadjuvant treatment of breast cancer. J Breast Cancer.

[4] Dieci MV, Radosevic-Robin N, Fineberg S, van den Eynden G, Ternes N, Penault-Llorca F (2017). International Immuno-Oncology Biomarker Working Group on Breast Cancer. Update on tumor-infiltrating lymphocytes (TILs) in breast cancer, including recommendations to assess TILs in residual disease after neoadjuvant therapy and in carcinoma in situ: A report of the International Immuno-Oncology Biomarker Working Group on Breast Cancer. Semin Cancer Biol.

[5] Pathak M, Dwivedi SN, Deo SVS, Thakur B, Sreenivas V, Rath GK (2018). Neoadjuvant chemotherapy regimens in treatment of breast cancer: a systematic review and network meta-analysis protocol. Syst Rev.

[6] Loibl S (2015). Neoadjuvant treatment of breast cancer: maximizing pathologic complete response rates to improve prognosis. Curr Opin Obstet Gynecol.

[7] Dodiya HG, Brahmbhatt AP, Khatri PK, Kaushal AM, Vijay DG (2015). Neoadjuvant chemotherapy in patients with locally advanced breast cancer: a pilot-observational study. J Cancer Res Ther.

[8] Smith IC, Heys SD, Hutcheon AW, Miller ID, Payne S, Gilbert FJ (2002). Neoadjuvant chemotherapy in breast cancer: significantly enhanced response with docetaxel. J Clin Oncol.

[9] Redana S, Sharp A, Lote H, Mohammed K, Papadimitraki E, Capelan M, Ring A (2016). Rates of major complications during neoadjuvant and adjuvant chemotherapy for early breast cancer: an off-study population. Breast.

[10] Nicolazzi MA, Carnicelli A, Fuorlo M, Scaldaferri A, Masetti R, Landolfi R (2018). Anthracycline and trastuzumab-induced cardiotoxicity in breast cancer. Eur Rev Med Pharmacol Sci.

[11] Houssami N, Macaskill P, von Minckwitz G, Marinovich ML, Mamounas E (2012). Meta-analysis of the association of breast cancer subtype and pathologic complete response to neoadjuvant chemotherapy. Eur J Cancer.

[12] Cortazar P, Geyer CE (2015). Pathological complete response in neoadjuvant treatment of breast cancer. Ann Surg Oncol.

[13] Salaroglio IC, Panada E, Moiso E, Buondonno I, Provero P, Rubinstein M (2017). PERK induces resistance to cell death elicited by endoplasmic reticulum stress and chemotherapy. Mol Cancer.

[14] Panaretakis T, Kepp O, Brockmeier U, Tesniere A, Bjorklund AC, Chapman DC (2009). Mechanisms of pre-apoptotic calreticulin exposure in immunogenic cell death. EMBO J.

[15] Mhaidat NM, Alali FQ, Matalqah SM, Matalka II, Jaradat SA, Al-Sawalha NA (2009). Inhibition of MEK sensitizes paclitaxel-induced apoptosis of human colorectal cancer cells by downregulation of GRP78. Anti-Cancer Drugs.

[16] Mandic A, Hansson J, Linder S, Shoshan MC (2003). Cisplatin induces endoplasmic reticulum stress and nucleus-independent apoptotic signaling. J Biol Chem.

[17] Yadunandam AK, Yoon JS, Seong YA, Oh CW, Kim GD (2012). Prospective impact of 5-FU in the induction of endoplasmic reticulum stress, modulation of GRP78 expression and autophagy in Sk-Hep1 cells. Int J Oncol.

[18] Pujari R, Jose J, Bhavnani V, Kumar N, Shastry P, Pal JK (2016). Tamoxifen-induced cytotoxicity in breast cancer cells is mediated by glucose-regulated protein 78 (GRP78) via AKT (Thr308) regulation. Int J Biochem Cell Biol.

[19] Almanza A, Carlesso A, Chintha C, Creedican S, Doultsinos D, Leuzzi B (2019). Endoplasmic reticulum stress signalling - from basic mechanisms to clinical applications. FEBS J.

[20] Li J, Lee AS (2006). Stress induction of GRP78/BiP and its role in cancer. Curr Mol Med.

[21] Sato M, Yao VJ, Arap W, Pasqualini R (2010). GRP78 signaling hub a receptor for targeted tumor therapy. Adv Genet.

[22] Kulasingam V, Prassas I, Diamandis EP (2017). Towards personalized tumor markers. NPJ Precis Oncol.

[23] Diamandis EP (2012). The failure of protein cancer biomarkers to reach the clinic: why, and what can be done to address the problem?. BMC Med.

[24] Mueller C, Haymond A, Davis JB, Williams A, Espina V (2018). Protein biomarkers for subtyping breast cancer and implications for future research. Expert Rev Proteomics.

[25] Grootjans J, Kaser A, Kaufman RJ, Blumberg RS (2016). The unfolded protein response in immunity and inflammation. Nat Rev Immunol.

[26] Raiter A, Yerushalmi R, Hardy B (2014). Pharmacological induction of cell surface GRP78 contributes to apoptosis in triple negative breast cancer cells. Oncotarget.

[27] Yerushalmi R, Raiter A, Nalbandyan K, Hardy B (2015). Cell surface GRP78: a potential marker of good prognosis and response to chemotherapy in breast cancer. Oncol Lett.

[28] Lee JH, Yoon YM, Lee SH (2017). Hypoxic preconditioning promotes the bioactivities of mesenchymal stem cells via the HIF-1α-GRP78-Akt axis. Int J Mol Sci.

[29] Lu MC, Lai NS, Yin WY, Yu HC, Huang HB, Tung CH (2013). Anti-citrullinated protein antibodies activated ERK1/2 and JNK mitogen-activated protein kinases via binding to surface-expressed citrullinated GRP78 on mononuclear cells. J Clin Immunol.

[30] Oida T, Weiner HL (2010). Overexpression of TGF-ß 1 gene induces cell surface localized glucose-regulated protein 78-associated latency-associated peptide/TGF-ß. J Immunol.

[31] Yao X, Liu H, Zhang X, Zhang L, Wang C, Sun S. Cell surface GRP78 accelerated breast cancer cell proliferation and migration by activating STAT3. PLoS One. 2015;10:1–17. 10.1371/journal.pone.0125634 PMC4431800 25973748

[32] Serrano-Negrón JE, Zhang Z, Rivera-Ruiz AP, Banerjee A, Romero-Nutz EC, Sánchez-Torres N (2018). Tunicamycin-induced ER stress in breast cancer cells neither expresses GRP78 on the surface nor secretes it into the media. Glycobiology.

[33] Bidard FC, Michiels S, Riethdorf S, Mueller V, Esserman LJ, Lucci A, et al. Circulating tumor cells in breast cancer patients treated by neoadjuvant chemotherapy: a meta-analysis. J Natl Cancer Inst. 2018;110:560–7. 10.1093/jnci/djy018. 10.1093/jnci/djy018 29659933

[34] Seidl M, Bader M, Vaihinger A, Wellner UF, Todorova R, Herde B, et al. Morphology of immunomodulation in breast cancer tumor draining lymph nodes depends on stage and intrinsic subtype. Sci Rep. 2018;8:5321. https://doi.org/10.1038/s41598-018-23629-3. 10.1038/s41598-018-23629-3 PMC5871837 29593307

[35] Cho HY, Thomas S, Golden EB, Gaffney KJ, Hofman FM, Chen TC (2009). Enhanced killing of chemo-resistant breast cancer cells via controlled aggravation of ER stress. Cancer Lett.

[36] Thaxton JE, Wallace C, Riesenberg B, Zhang Y, Paulos CM, Beeson CC (2017). Modulation of endoplasmic reticulum stress controls CD4^+^ T-cell activation and antitumor function. Cancer Immunol Res.

[37] Brinkman CC, Peske JD, Engelhard VH (2013). Peripheral tissue homing receptor control of naïve, effector, and memory CD8 T cell localization in lymphoid and non-lymphoid tissues. Front Immunol.

[38] Appay V, Dunbar PR, Callan M, Klenerman P, Gillespie GM, Papagno L (2002). Memory CD81 T cells vary in differentiation phenotype in different persistent virus infections. Nat Med.

[39] Månsson Kvarnhammar A, Uddman R, Björnsson S, Riesbeck K, Cardell LO (2012). The activation pattern of blood leukocytes in head and neck squamous cell carcinoma is correlated to survival. PLoS One.

[40] Seyfizadeh N, Muthuswamy R, Mitchell DA, Nierkens S, Seyfizadeh N (2016). Migration of dendritic cells to the lymph nodes and its enhancement to drive anti-tumor responses. Crit Rev Oncol Hematol.

[41] Muraro E, Comaro E, Talamini R, Turchet E, Miolo G, Scalone S (2015). Improved natural killer cell activity and retained anti-tumor CD8(+) T cell responses contribute to the induction of a pathological complete response in Her2-positive breast cancer patients undergoing neoadjuvant chemotherapy. J Transl Med.

[42] Vig S, Buitinga M, Rondas D, Crèvecoeur I, van Zandvoort M, Waelkens E (2019). Cytokine-induced translocation of GRP78 to the plasma membrane triggers a pro-apoptotic feedback loop in pancreatic beta cells. Cell Death and Disease.

[43] Smith JA (2018). Regulation of cytokine production by the unfolded protein response; Implications for Infection and Autoimmunity. Front Immunol.

[44] Zaidi MR (2019). The interferon-gamma paradox in Cancer. J Interf Cytokine Res.

[45] Galluzzi L, Zitvogel L, Kroemer G (2016). Immunological mechanisms underneath the efficacy of Cancer therapy. Cancer Immunol Res.

[46] Ma Y, Ren Y, Dai ZJ, Wu CJ, Ji YH, Xu J (2017). IL-6, IL-8 and TNF-α levels correlate with disease stage in breast cancer patients. Adv Clin Exp Med.

[47] Jin K, Pandey NB, Popel AS (2018). Simultaneous blockade of IL-6 and CCL5 signaling for synergistic inhibition of triple-negative breast cancer growth and metastasis. Breast Cancer Res.

[48] Garg AD, Kaczmarek A, Krysko O, Vandenabeele P, Krysko DV, Agostinis P (2012). ER stress-induced inflammation: does it aid or impede disease progression?. Trends Mol Med.

