# Supplementary material for: GRP78 expression in peripheral blood mononuclear cells is a new predictive marker for the benefit of taxanes in breast cancer neoadjuvant treatment
Source: BMC Cancer. 2020 Apr 19;20:333. doi: 10.1186/s12885-020-06835-z (PMC7168854; doi:10.1186/s12885-020-06835-z)
Supplement: Supplementary file 1 — Additional file 1: Table S1. Percentage T, NK and monocytes sub-populations before and after chemotherapy. [file 12885_2020_6835_MOESM1_ESM.docx]

| **PBMCs**  **sub-populations** | **P1** | **P3** | **P1** | **P3** |
| --- | --- | --- | --- | --- |
|  | **pCR (N=20)** | | **Non pCR (N=6)** | |
| CD3 | 58.9±10.3 | 61±5.4 | 49.8±13.2 | 51.1±11.2 |
| CD4 | 48.4+15.5 | 47.7±6.1 | 38±9.8 | 41.6±7.2 |
| CD8 | 22.7±8.9 | 19.1±7.4 | 21.8 | 19.3±10.2 |
| CD3/CD62L | 13.9±7.9 | 11.7±9.2 | 11.7±5.4 | 12.5±7.1 |
| CD3/CCR7 | 9.1±6.0 | 9.4±8.9 | 8.7±4.5 | 9.1±3.9 |
| CD3/CD56 | 8.3±6.5 | 10.3±4.2 | 8.8±3.9 | 9.8±4.2 |
| CD8/CD56 | 4.3±4.8 | 5.6±4.1 | 4.4±4.4 | 4.9±3.6 |
| CD56 | 9.3±6.9 | 9.9±6.8 | 9.1±4.6 | 8.1±6.8 |
| NKG2D | 5.1±4.2 | 3.8±2.1 | 5.2±3.5 | 4.6±3.7 |
| CD56/NKG2D | 3.6±3.1 | 4.2±2.9 | 4.1±3.4 | 4.7±2.9 |
| CD16 | 9.1±7.2 | 8.5±7.3 | 8.5±6.4 | 8.1±7.3 |
| CD45RA | 24.7±11.9 | 19.7±10.3 | 20.2±6.1 | 21.5±8.8 |
| CD45RO | 14.5±9.2 | 20.1±8.5 | 11.7±7.3 | 17.7±9.1 |
| CD14 | 8.8±7.7 | 7.2±5.7 | 7.9±6.3 | 6.9±5.1 |
| CD14/CD62L | 5.3±3.6 | 5.3±4.1 | 4.8±4.7 | 5.1±3.9 |

**Table S1:**

**Percentage T, NK and monocytes sub-populations before and after chemotherapy.**

The percentage of the different sub-populations was evaluated by FACS analysis at baseline (P1) and just before commencing the paclitaxel phase (P3).
